# Serum lipoprotein(a) and risk of periprocedural myocardial injury in patients undergoing percutaneous coronary intervention

**DOI:** 10.1002/clc.23520

**Published:** 2020-12-02

**Authors:** Zhuoshan Huang, Xing Shui, Yesheng Ling, Linli Zhou, Wenqi Shi, Yanting Luo, Suhua Li, Jieming Zhu, Shujie Yu, Jinlai Liu

**Affiliations:** ^1^ Department of Cardiovascular Medicine The Third Affiliated Hospital, Sun Yat‐sen University Guangzhou China; ^2^ Mental and Neurological Diseases Research Center Office The Third Affiliated Hospital, Sun Yat‐sen University Guangzhou China; ^3^ Medical Records Management Office The Third Affiliated Hospital, Sun Yat‐sen University Guangzhou China

**Keywords:** high‐sensitivity cardiac troponin I, lipoprotein(a), percutaneous coronary intervention, periprocedural myocardial injury

## Abstract

Recent studies and guidelines have indicated that lipoprotein(a) [Lp(a)]was an independent risk factor of arteriosclerotic cardiovascular disease (ASCVD). This study aimed to determine the relationship between serum Lp(a) levels and the risk of periprocedural myocardial injury following percutaneous coronary intervention (PCI) in coronary heartdisease (CHD) patients. This study enrolled 528 nonacute myocardial infarction (AMI) coronary heart disease (CHD) patients who successfully underwent PCI. Fasting serum lipids including Lp(a) were tested before PCI. High‐sensitivity cardiac troponin I (hs‐cTnI) was tested before PCI and 24 h after PCI. Univariate and multivariate logistic regression analyses were used to determine the relationship between preprocedural Lp(a) levels and postprocedural cTnI elevation from 1 × upper limit of normal (ULN) to 70 × ULN. As a continuous variable, multivariate analyses adjusting for conventional covariates and other serum lipids revealed that increased Lp(a) levels were independently associated with the risk of elevated postprocedural cTnI values above 1 × ULN (odds ratio [OR] per log‐unit higher: 1.31, 95% confidence interval [CI]: 1.02–1.68, P = 0.033], 5 × ULN (OR: 1.25, 95%CI: 1.02–1.53, P = 0.032), 10 × ULN (OR: 1.48, 95%CI: 1.18–1.86, P = 0.001) and 15 × ULN (OR: 1.28, 95%CI: 1.01–1.61, P = 0.038). As a categorical variable, Lp(a) > 300 mg/L was an independent risk factor of postproceduralc TnI≥1 × ULN (OR 2.17, 95%CI 1.12–4.21, P = 0.022), ≥5 × ULN (OR 1.82, 95%CI 1.12–2.97, P = 0.017) and ≥10 × ULN (OR 2.17, 95%CI 1.33–3.54, P = 0.002). Therefore, it could be concluded that elevated preprocedural Lp(a) levels were associated with the risk of PCI‐related myocardial injury in non‐AMI CHD patients.

## 1 INTRODUCTION

Lipoprotein(a) [Lp(a)] is a lipoprotein composed of low‐density lipoprotein (LDL) and an additional protein apolipoprotein(a).^1^ It is a member of human plasma lipoproteins which also include chylomicrons, very low‐density lipoprotein (VLDL), intermediate‐density lipoprotein (IDL), LDL and high‐density lipoprotein (HDL).^2^ LDL consisting of cholesterol and apoB‐100 delivers cholesterol from liver to peripheral tissues.^3^ For decades, low‐density lipoprotein cholesterol (LDL‐C) has already been proven to be the most important risk factor atherosclerosis cardiovascular disease (ASCVD).^2^, ^4^, ^5^, ^6^, ^7^ Different to LDL, an additional apolipoprotein(a) [apo(a)] makes the physiological and vascular effects of Lp(a) ambiguous.^1^ However, uncertain mechanism as it was, studies have already reported the positive correlation between plasma Lp(a) levels and the risk of ASCVD.^8^, ^9^ Recent ESC/EAS guidelines for the management of dyslipidaemias recommended at least once measurement of Lp(a) in the lifetime to identify the persons who may have a lifetime risk of ASCVD.^2^

Percutaneous coronary intervention (PCI) is the major therapeutic strategy of coronary heart disease (CHD). Myocardial infarction (MI) associated with PCI which was classified as type 4a MI in Fourth Universal Definition of Myocardial Infarction was one of the major complications of PCI.^10^ Previous studies have already confirmed that type 4a MI would cause a poor prognosis after PCI.^11^, ^12^, ^13^ Different to type 4a MI, elevation of cardiac troponin (cTn) after PCI which was defined as PCI‐related myocardial injury was relatively common especially after the use of high‐sensitivity cTn (hs‐cTn).^14^ Although the optimal cut‐off value of postprocedural cTn which was of prognostic significance was still controversial, our previous study revealed that the higher the postprocedural cTnI, the worse the prognosis of patients.^15^ The relationship between LDL‐C, high‐density lipoprotein cholesterol (HDL‐C) or non‐HDL‐C and different levels of postprocedural cTn elevation have been studied before.^16^, ^17^, ^18^ However, whether higher Lp(a) levels were associated with risk of PCI‐related myocardial injury remained unknown. Thus, this study sought to explore the association between preprocedural serum Lp(a) levels and PCI‐related myocardial injury. Moreover, as mentioned above, the cut‐off value of postprocedural cTnI with prognostic significance was controversial yet, therefore we investigated the association between Lp(a) and different elevations of postprocedural cTnI levels from 1 × ULN up to 70 × ULN.

## 2 METHOD

### 2.1 Study population

**FIGURE 1:**
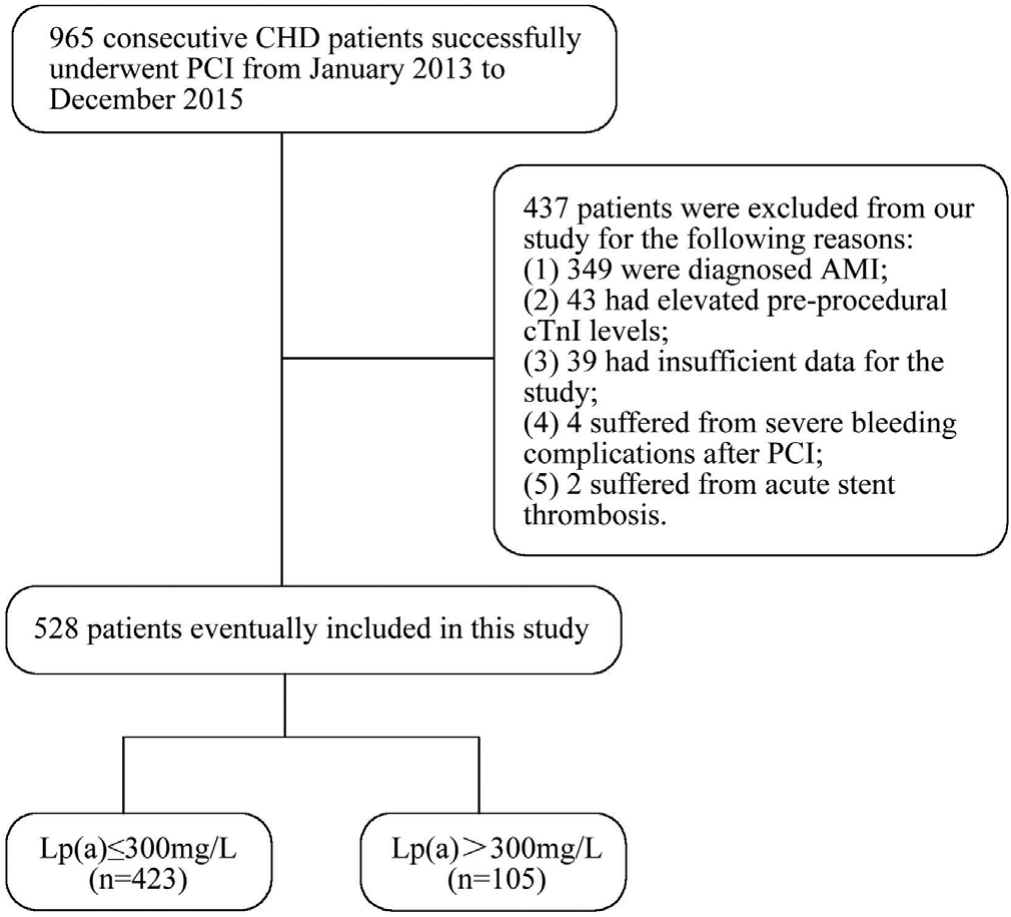
Flowchart illustrating study population. CHD, coronary heart disease; PCI, percutaneous coronary intervention; AMI, acute myocardial infarction

This is a multicenter retrospective study. CHD patients who successfully underwent PCI at our hospital were screened for eligibility. Success of PCI was defined as residual stenosis <20% with stenting by visual estimation. Exclusion criteria included: (a) acute myocardial infarction (AMI) occurred within 8 weeks before PCI; (b) cTnI level was higher than ULN before PCI; (c) severe bleeding complications such as cerebral hemorrhage or gastrointestinal hemorrhage occurred after PCI; (d) acute stent thrombosis occurred within 24 h after PCI which caused type 4b MI^10^; (e) use of proprotein convertase subtilisin/kexin type 9 (PCSK9) inhibitors or nicotinic acid which could reduce the Lp(a) levels^2^, ^19^; (f) insufficient clinical data. From January 2013 to December 2015, a total of 965 consecutive CHD patients successfully underwent PCI at the Third Affiliated Hospital of Sun Yat‐sen University and the Third Affiliated Hospital of Sun Yat‐sen University‐Lingnan Hospital. Of these patients, 349 patients were diagnosed AMI and excluded from our study. Besides, 43 patients were excluded because of elevated preprocedural cTnI levels, 39 patients were excluded because their clinical data available were insufficient for the study, while four and two patients were excluded respectively because of severe bleeding complications and acute stent thrombosis after PCI. Eventually, 528 patients were included in the present study (Figure 1). The current study was approved by the Institutional Review Board of the hospital.

### 2.2 Measurement of laboratory data

After admission, fasting blood samples were obtained from each patient before the procedure. Serum lipids including total cholesterol (TC), triglyceride (TG), LDL‐C, HDL‐C, apoAI, apoB100 and Lp(a) were determined by Hitachi 7600 automatic biochemical analyzer. LDL‐C and HDL‐C were analyzed using the direct method. TC and TG were analyzed using enzyme colorimetry method. Lp(a) was determined by latex immunoturbidimetry method. ApoAI and apoB100 were determined by immunoturbidimetry method. Non‐HDL‐C levels were calculated from TC minus HDL‐C levels. Hs‐cTnI was analyzed by chemiluminescence method through Abbott ARCHITECT i2000SR chemiluminescence immunoanalyzer. Upper limit of normal (ULN) which was defined as the 99th percentile of normal population was used to replace upper reference limit (URL). ULN of hs‐cTnI was 0.04 ng/ml of our test. Blood samples were obtained before PCI and 24 h after PCI to determine preprocedural and postprocedural cTnI levels.

### 2.3 Coronary angiography and percutaneous coronary intervention

Each patient received sufficient dose of aspirin and clopidogrel before procedure (aspirin 0.1 g daily for at least 3 days or a loading dose of 0.3 g, clopidogrel 75 mg daily for at least 4 days or a loading dose of 300 mg). Unless there were absolute contraindications, all patients received moderate‐intensity statin therapy (atorvastatin 20 mg/d or rosuvastatin 10 mg/d) before procedure. Patients underwent coronary angiography (CAG) firstly and PCI indication was decided by two experienced interventional cardiologists according to Chinese guideline for percutaneous coronary interventional.^20^ Severity of coronary artery lesion was evaluated by Gensini Score.^21^ Firstly, concentric and eccentric stenoses were assigned a corresponding numeric value from 1 to 100% obstruction. The value was then multiplied by a coefficient corresponding to location of stenosis. Gensini score was summation of all products of stenosis extent and location of stenosis. PCI was performed by experienced interventional cardiologists. Patients received 100 U/kg bolus unfractionated heparin (UFH) just before the procedure and an additional bolus of 1000 U was given every hour if the procedure lasted for >1 h. Activated clotting time (ACT) was measured 1 h after procedure finished to make clear the response of UFH for each patient. Parameters of PCI including intervention vessels, number of stents, total stent length, balloon expansion pressure after procedure and intra‐operative complications were recorded. All stents were drug‐eluting stents (DES).

### 2.4 Statistical analysis

**FIGURE 2:**
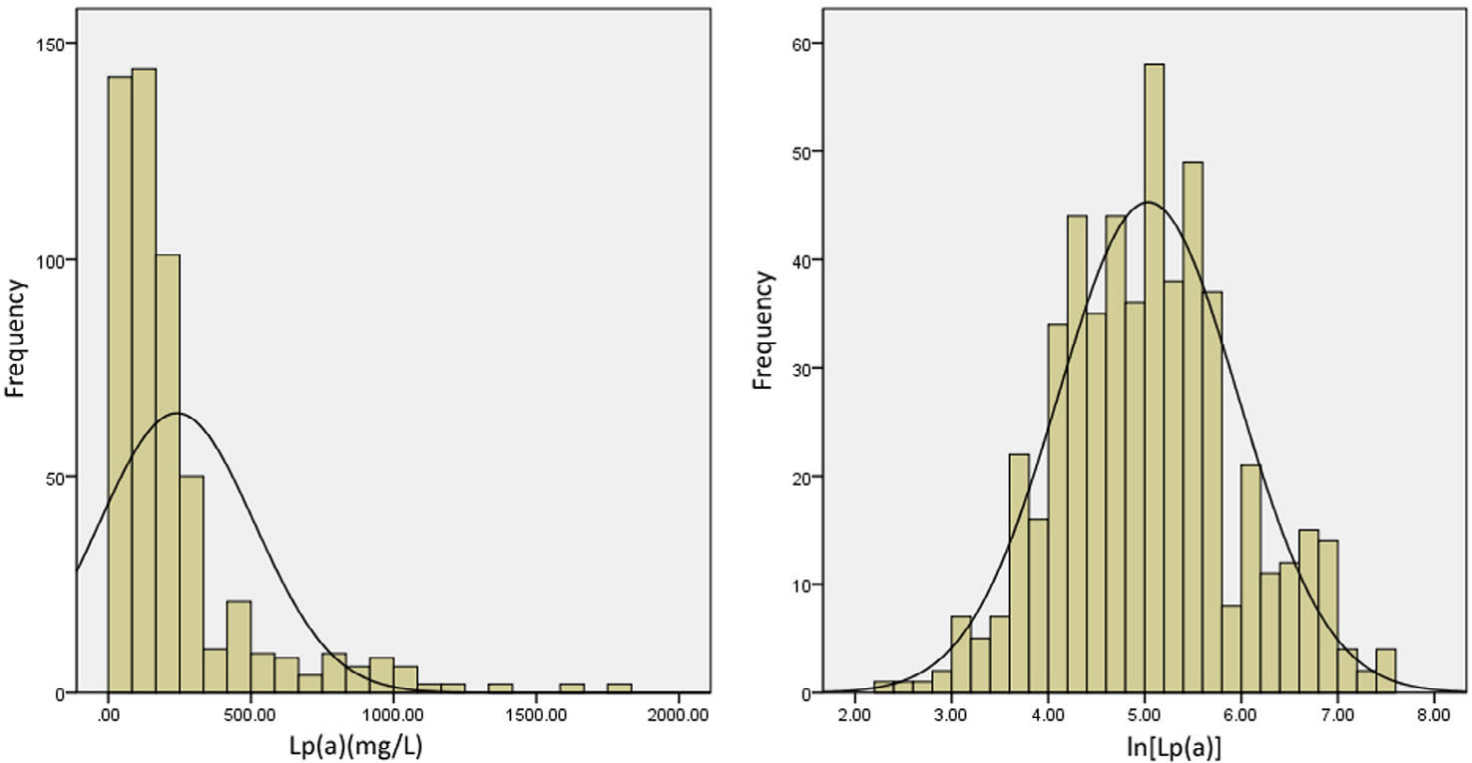
Distribution of Lp(a) levels and ln[Lp(a)] levels in the study population

Continuous data were expressed as mean ± SD and categorical data were presented as frequencies with percentage. Student's t‐test or one‐way analysis of variance was performed to determine the differences in continuous data between groups and chi‐square test was performed for categorical variables. Logistic regression analyses were performed to explore the relationship between Lp(a) and different times ULN of postprocedural cTnI elevations. Univariate logistic regression analyses were performed firstly. Lp(a) was respectively analyzed as a continuous variable or a categorical variable with a cut‐off value as 300 mg/L according to Guidelines for the Prevention and Treatment of Dyslipidemia in Chinese Adults.^22^ Since the distribution of Lp(a) showed positive skewing (Figure 2), it was logarithmically transformed when analyzed as a continuous variable and the results of logistic regression analysis were represented by odds ratio (OR) per log‐unit increase. Multivariate logistic regression analyses were performed in two different models. Model 1 was adjusted for conventional covariates excluding other serum lipids. In model 2, we added TG, LDL‐C, non‐HDL‐C, HDL‐C, apoAI, apoB100 as variables for the purpose of assessing the independent association of Lp(a) and PCI‐related myocardial injury. Variables selection of multivariate logistic regression models was done by stepwise forward method and threshold values for F‐to‐enter and F‐to‐remove were 0.05 and 0.1, respectively. All analyses were performed using Statistical Program for Social Sciences (SPSS), version 22.0, software (Chicago, IL). *p*‐value of <0.05 was considered statistically significant.

## 3 RESULTS

### 3.1 Baseline characteristics and procedure parameters

A total of 528 patients were included in our study. The baseline serum Lp(a) levels ranged from 11 to 1797 mg/L (median = 153 mg/L, interquartile range = 80‐265 mg/L) (Figure 2). Patients were divided into two groups according to Lp(a) ≤ 300 mg/L and > 300 mg/L. Several baseline characteristics were compared between two groups (Table 1). Patients with lower Lp(a) levels were older, more likely to have diabetes, higher BMI, lower NT‐proBNP and creatine levels. CHD drug therapies were not significantly different between two groups. As for serum lipids, patients with lower Lp(a) levels also had lower TC, LDL‐C, HDL‐C, non‐HDL‐C levels while TG levels were of no difference. CAG and PCI parameters were shown in Supplementary Table 1. There were no significant differences between two groups in terms of procedure parameters.

**TABLE 1:** Baseline characteristics

Variable	Lp(a) ≤ 300 mg/L (n = 423)	Lp(a)>300 mg/L (n = 105)	*p* value
Male, n (%)	291 (69.9)	74 (66.7)	0.527
Age, y	67.51 ± 10.32	63.88 ± 9.40	0.001*
BMI, kg/m^2^	24.52 ± 3.27	23.73 ± 3.27	0.029*
Hypertension, n (%)	306 (73.4)	87 (78.4)	0.283
Diabetes, n (%)	154 (36.9)	26 (23.4)	0.008*
Prior PCI, n (%)	54 (12.9)	16 (14.4)	0.686
Smoking, n (%)	143 (34.3)	41 (36.9)	0.603
Family history of CHD, n (%)	36 (8.6)	11 (9.9)	0.675
Unstable angina, n (%)	117 (28.1)	32 (28.8)	0.873
Hemoglobin (g/L)	135.59 ± 15.56	134.70 ± 16.81	0.607
TC (mmol/L)	4.27 ± 1.19	4.96 ± 1.43	<0.001*
TG (mmol/L)	1.78 ± 1.74	1.60 ± 0.72	0.288
LDL‐C (mmol/L)	2.60 ± 0.95	3.22 ± 1.27	<0.001*
HDL‐C (mmol/L)	1.03 ± 0.25	1.15 ± 0.37	<0.001*
non‐HDL‐C (mmol/L)	3.25 ± 1.11	3.82 ± 1.50	<0.001*
apoAI (g/L)	1.26 ± 0.20	1.28 ± 0.25	0.382
apoB100 (g/L)	1.15 ± 0.75	1.21 ± 0.35	0.400
Creatine (umol/L)	88.22 ± 25.48	104.56 ± 134.03	0.019*
Uric Acid (umol/L)	411.73 ± 112.84	392.70 ± 103.68	0.117
Glycosylated hemoglobin (%)	6.39 ± 1.24	6.37 ± 1.38	0.908
HsCRP (mg/L)	5.68 ± 12.66	3.93 ± 6.02	0.255
NT‐proBNP^a^			0.018*
normal, n (%)	359 (86.1)	84 (75.7)	
gray zone, n (%)	38 (8.6)	14 (12.6)	
high, n (%)	22 (5.3)	13 (11.7)	
LVEF<50%, n (%)	36 (8.6)	14 (12.6)	0.203
E/A ratio < 0.8, n (%)	179 (42.9)	42 (37.8)	0.334
MAP^b^ (mmHg)	95.86 ± 10.98	95.97 ± 11.15	0.926
Baseline cTnI levels (ng/ml)	0.009 ± 0.009	0.007 ± 0.008	0.073
Drug therapies			
ACEI/ARB, n (%)	333 (78.7)	91 (86.7)	0.067
β‐blocker, n (%)	200 (48.0)	50 (45.0)	0.584
CCB, n (%)	175 (42.0)	52 (46.8)	0.356
Trimetazidine, n (%)	256 (61.4)	65 (58.6)	0.587
Nitrates, n (%)	161 (38.6)	47 (42.3)	0.474
Statin intensity^c^			0.542
Moderate‐intensity, n (%)	415 (98.1)	101 (96.2)	
Low‐intensity, n (%)	6 (1.4)	3 (2.8)	
No statins, n (%)	2 (0.5)	1 (1.0)	

Abbreviations: BMI, body mass index; HsCRP, high‐sensitivity C‐reactive protein; MAP, mean arterial pressure; ACEI, angiotensin converting enzyme inhibitor; ARB, angiotensin receptor blocker; CCB, calcium channel blocker.

^a^ NT‐proBNP was calculated using the following classification: “normal” was <300 pg/ml; “gray zone” was 300–450 pg/ml for<50 years, 300–900 pg/ml for 50–75 years and 300–1800 pg/ml for >75 years; “high” was >450 pg/ml for <50 years, >900 pg/ml for 50–75 years and > 1800 pg/ml for >75 years.

^b^ MAP = 1/3*(systolic pressure) + 2/3*(diastolic pressures).

^c^ Moderate‐intensity statin therapy: atorvastatin 20 mg/d, rosuvastatin 10 mg/d. Low‐intensity statin therapy: fluvastatin 40 mg/d, simvastatin 10 mg/d, pravastatin 20 mg/d.

* *p* < 0.05.

### 3.2 Association between Lp(a) levels and postprocedural cTnI levels

**FIGURE 3:**
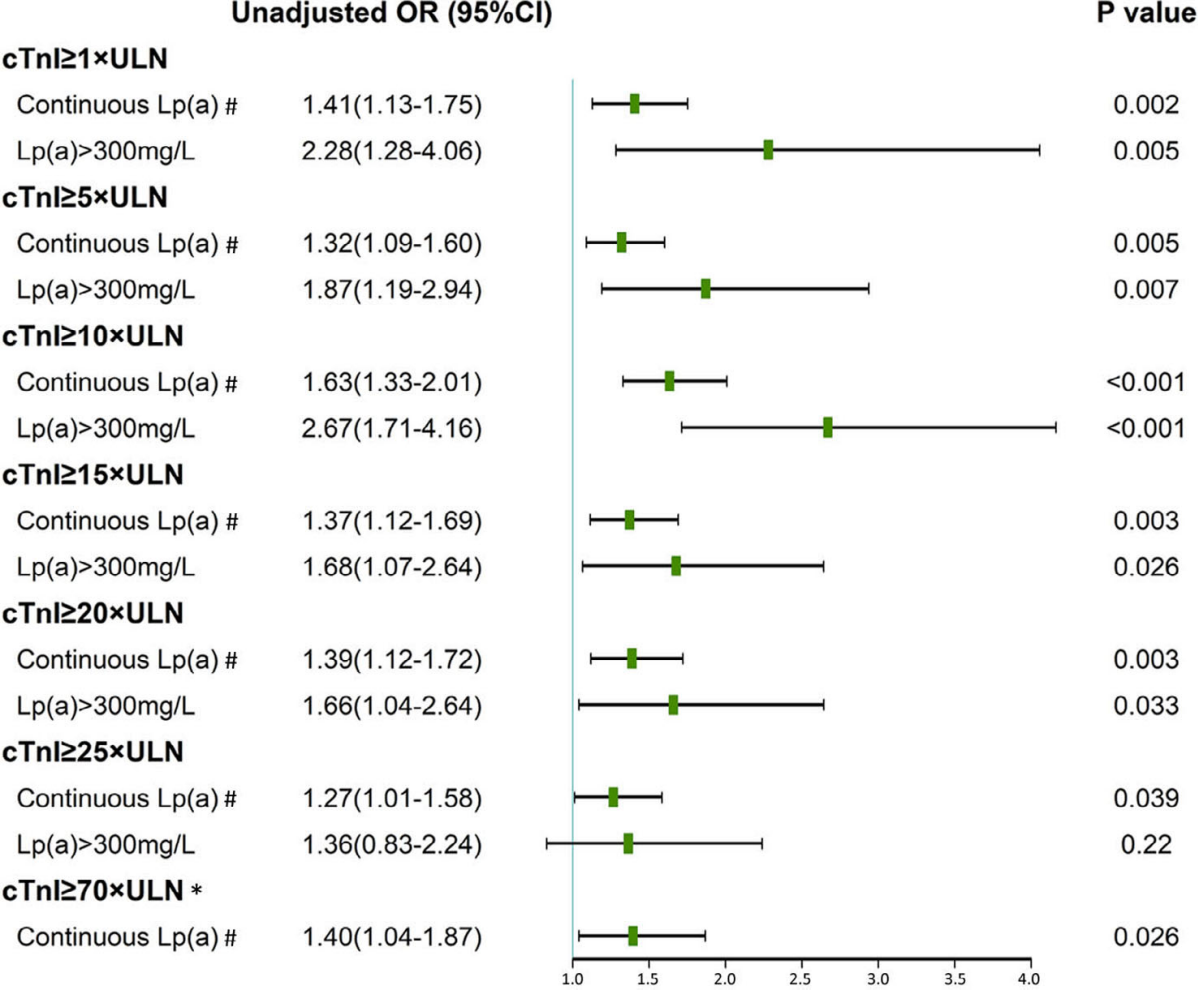
Univariate logistic regression analysis results of correlation between preprocedural serum Lp(a) and postprocedural cTnI elevations. OR, odds ratio; ULN, upper limit of normal. #Results of continuous Lp(a) were represented by OR per log‐unit increase. *The association between Lp(a) > 300 mg and postprocedural cTnI≥70 × ULN was not investigated because of the small sample size

Postprocedural cTnI ≥1 × ULN, ≥5 × ULN, ≥10 × ULN, ≥25 × ULN, ≥70 × ULN was detected in 321 (60.8%), 166 (31.4%), 114 (21.6%), 76 (14.4%), and 45 (8.5%) patients, respectively. As a continuous variable, univariate logistic regression analyses revealed that increasing Lp(a) level was positively correlated to risk of each postprocedural cTnI elevation (Figure 3).

After adjustment of conventional covariates excluding other serum lipids in model 1, increasing Lp(a) level was positively correlated to risk of postprocedural cTnI ≥1 × ULN, ≥5 × ULN, ≥10 × ULN, ≥15 × ULN, ≥20 × ULN but not cTnI ≥25 × ULN and ≥ 70 × ULN. In model 2 including other serum lipids as covariates together, increasing Lp(a) level was still positively correlated to risk of postprocedural cTnI greater than 1 × ULN up to 15 × ULN, while it was not correlated to cTnI ≥20 × ULN, 25 × ULN or 70 × ULN (Table 2). For comparison, adjusted OR per 1 mmol/L increment of LDL‐C in model 2 was also shown in Table 2. Increasing LDL‐C level was positively correlated to risk of postprocedural cTnI greater than 1 × ULN up to 20 × ULN. Of cTnI ≥25 × ULN and ≥ 70 × ULN, none of serum lipids was found relevant, while LVEF<50%, Gensini Scores, total stent length, intra‐operative complications were found positively relevant and hemoglobin was found negatively (Supplementary Figure 1).

**TABLE 2:** Multivariate Logistic Regression Analyses Results of the Correlation between Preoperative Lp(a) and Postoperative cTnI

	Model 1^a^		Model 2^b^	
	OR (95%CI)	*p* value	OR (95%CI)	*p* value
**cTnI ≥ 1 × ULN**				
Continuous Lp(a)^c^	1.38 (1.08–1.76)	0.011	1.31 (1.02–1.68)	0.033*
Lp(a)>300 mg/L	2.56 (1.36–4.83)	0.004	2.17 (1.12–4.21)	0.022*
Continuous LDL‐C^d^	—	—	1.56 (1.22‐2.01)	<0.001*
**cTnI ≥ 5 × ULN**				
Continuous Lp(a)^c^	1.29 (1.05–1.59)	0.014	1.25 (1.02–1.53)	0.032*
Lp(a)>300 mg/L	1.90 (1.17–3.08)	0.009	1.82 (1.12–2.97)	0.017*
Continuous LDL‐C^d^	—	—	1.63 (1.34‐1.98)	<0.001*
**cTnI ≥ 10 × ULN**				
Continuous Lp(a)^c^	1.62 (1.29–2.03)	<0.001	1.48 (1.18–1.86)	0.001*
Lp(a)>300 mg/L	2.56 (1.59–4.12)	<0.001	2.17 (1.33–3.54)	0.002*
Continuous LDL‐C^d^	—	—	1.56 (1.27‐1.91)	<0.001*
**cTnI ≥ 15 × ULN**				
Continuous Lp(a)^c^	1.33 (1.06–1.67)	0.013	1.28 (1.01–1.61)	0.038*
Lp(a)>300 mg/L	1.66 (1.03–2.69)	0.039	1.26 (0.76–2.10)	0.369
Continuous LDL‐C^d^	—	—	1.45 (1.17‐1.79)	0.001*
**cTnI ≥ 20 × ULN**				
Continuous Lp(a)^c^	1.33 (1.04–1.69)	0.016	1.24 (0.98–1.58)	0.080
Lp(a)>300 mg/L	1.48 (0.89–2.47)	0.134	1.28 (0.76–2.16)	0.359
Continuous LDL‐C^d^	—	—	1.32 (1.07‐1.64)	0.011*
**cTnI ≥ 25 × ULN**				
Continuous Lp(a)^c^	1.22 (0.95–1.57)	0.123	1.16 (0.90–1.51)	0.260
Lp(a)>300 mg/L	1.15 (0.66–2.00)	0.630	1.04 (0.59–1.84)	0.891
Continuous LDL‐C^d^	—	—	1.20 (0.95‐1.50)	0.127
**cTnI ≥ 70 × ULN**				
Continuous Lp(a)^c^	1.41 (0.98–2.01)	0.063	1.35 (0.94–1.93)	0.103
Continuous LDL‐C^d^	—	—	1.19 (0.93‐1.52)	0.171

^a^ Multivariate Model 1 adjusted for gender, age, BMI, type of CHD (unstable angina or not), diabetes, hypertension, smoking history, prior PCI history, family history of CAD, hemoglobin, creatinine, uric acid, glycosylated hemoglobin, hsCRP, NT‐proBNP, LVEF<50%, E/A ratio < 0.8, MAP, drugs, Gensini Score, number of intervention vessels, intervention vessel location, number of stents, total stent length, maximum inflation pressure, ACT 1 h after PCI finished, intra‐operative complication.

^b^ Multivariate Model 2 adjusted for covariates in Model 1 and LDL‐C, HDL‐C, non‐HDL‐C, TG, apoAI, apoB100.

^c^ Per log‐unit increment in Lp(a) levels.

^d^ Per mmol/L increment in LDL‐C levels analyzed in models with continuous Lp(a).

* *p* < 0.05.

### 3.3 Association of Lp(a) categories with postprocedural cTnI elevation

Univariate and multivariate logistic regression analyses were also performed to determine the association of Lp(a) > 300 mg and postprocedural cTnI elevation. As the small sample size of patients with both Lp(a) > 300 mg and cTnI≥70 × ULN, we have not investigated the association between Lp(a) > 300 mg and postprocedural cTnI≥70 × ULN. Univariate analyses found Lp(a) > 300 mg was positively correlated to risk of postprocedural cTnI≥1 × ULN, ≥5 × ULN, ≥10 × ULN, ≥15 × ULN, ≥20 × ULN but not ≥25 × ULN (Figure 3). In model 1 adjusting for conventional covariates excluding other serum lipids, Lp(a) > 300 mg was correlated to risk of postprocedural cTnI greater than 1 × ULN up to 15 × ULN, while it was not relevant to cTnI ≥20 × ULN and ≥ 25 × ULN. After adjusting for covariates including serum lipids, Lp(a) > 300 mg was still associated with postprocedural cTnI≥1 × ULN, ≥5 × ULN, ≥10 × ULN. Patients with Lp(a) > 300 mg were 1.2, 0.8, 1.2 times more likely to have postprocedural cTnI≥1 × ULN, ≥5 × ULN, ≥10 × ULN comparing to those with Lp(a) ≤ 300 mg, respectively. In this model, however, there were no relationships between Lp(a) > 300 mg/L and cTnI greater than 15 × ULN, 20 × ULN, 25 × ULN. (Table 2).

## 4 DISCUSSION

Our study demonstrated the positive correlation of preprocedural Lp(a) levels and postprocedural cTnI levels in non‐AMI CHD patients undergoing elective PCI, indicating that Lp(a) was an independent risk factor of PCI‐related myocardial injury.

For these years, the status of Lp(a) in risk of ASCVD have been receiving increasing attention. As we know, cigarette smoking, diabetes, hypertension and elevated LDL‐C level were the major risk factors promoting ASCVD.^23^ However, after controlling these risk factors, cardiovascular events still occurred in some people. Therefore, other factors correlating to ASCVD have been being explored for decades, which called residual cardiovascular risk.^24^ Lp(a) was considered to be a useful marker for identifying and evaluating the residual cardiovascular risk.^25^ Although the first epidemiological study was conducted by Dahlén G et al in 1972,^26^ the studies investigating the association between Lp(a) and cardiovascular disease were obstructed as the unclear mechanism and nonstandard measurement approaches of this lipoprotein. Hence, Lp(a) was rarely mentioned in the earlier guidelines about serum lipids.^27^, ^28^, ^29^ Fortunately, the First World Health Organization (WHO)/International Federation of Clinical Chemistry and Laboratory Medicine (IFCC) International Reference Reagent for Lp(a) for Immunoassay emerged in 2000 and was accepted by WHO in 2003.^30^, ^31^ Afterwards, a large scale meta‐analysis including 126 634 participants in 36 prospective studies revealed the continuous, independent and modest associations of Lp(a) levels with risk of CHD and ischemic stroke.^32^ Furthermore, Lp(a) levels were found largely genetically determined by the LPA gene,^33^ making mendelian randomization analyses demonstrating the relationship of Lp(a) and CHD feasible.^34^, ^35^, ^36^ The most recent mendelian randomization analysis including 13 781 individuals from the Lp(a)‐GWAS‐Consortium from five primarily population‐based studies and 20 793 CHD cases and 27 540 controls from a subsample of the CHD Exome+ consortium estimated a required reduction in Lp(a) effect size of 65.7 mg/dL to reach the same effect as a 1 mmol/L lowering of LDL‐C.^37^

Highly related as it was between lipids and CHD, previous studies have also focused on the relationship between serum lipids and type 4a MI or PCI‐related myocardial injury. Elevated LDL‐C or non‐HDL‐C levels have been found to be positively correlated to postprocedural cTnI levels.^16^ Another study considered non‐HDL‐C was more valuable in predicting PCI‐related myocardial injury than LDL‐C in type 2 diabetes patients.^18^ Moreover, higher HDL‐C levels were reported to be associated with less risk of PCI‐related myocardial injury in patients with LCL‐C < 70 mg/dL.^17^ Our previous study^15^ exploring the risk factors of type 4a MI found LDL‐C was an independent risk factor and 1‐SD increment of LDL‐C (1.05 mmol/L in the study) increased the risk of type 4a MI by 44%. In the study we have already noticed that Lp(a) levels seemed to be higher in patients with type 4a MI but the difference was not statistically significant. Therefore, we expanded the sample size and finished our present study. In this study, we analyzed 528 non‐AMI CHD patients and finally we found elevating Lp(a) was associated with risk for postprocedural cTnI levels above 1 × ULN up to 15 × ULN after adjustment of covariates including other serum lipids, indicating that Lp(a) was associated to PCI‐related myocardial injury independently of other serum lipids.

Definition and prognostic significance of PCI‐related myocardial infarction or injury have been developing for these years.^10^, ^38^, ^39^, ^40^ The preferred biomarker has been identified as cTn rather than CK‐MB^10^, ^38^ considering to the better sensitivity and specificity. Among the assays, high‐sensitivity cTn was more recommended.^41^ According to the most recent universal definition of MI,^10^ in patients with normal preprocedural cTn level, PCI‐related MI was defined as elevation of postprocedural cTn levels more than 5 × ULN with evidence of new myocardial ischemia. As mentioned above, PCI‐related MI would cause a poor prognosis after PCI.^11^, ^12^, ^13^ Unlike PCI‐related MI, elevation of cTn values after PCI which was arbitrarily defined as PCI‐related myocardial injury was of controversially significance.^11^, ^39^, ^42^, ^43^, ^44^, ^45^, ^46^ In our previous study,^15^ we found patients with postprocedural cTnI levels≥10 × ULN had a higher incidence of major adverse cardiovascular events (MACE) in 3 years after PCI comparing to those with normal postprocedural cTnI levels, while postprocedural cTnI levels from 1 × ULN up to 10 × ULN seemed to be no prognostic significance. Zeitouni et al found patients with postprocedural cTnT from 1 × ULN up to 5 × ULN with evidence of ischemia or cTnT≥5 × ULN without ischemic findings who could be diagnosed as periprocedural myocardial injury according to the Third universal definition of MI had an increased rate of cardiovascular events at 30 days and 1 year.^11^ While Ndrepepa et al found elevation of postprocedural cTnT levels did not offer prognostic information.^46^ Another expert consensus proposed by the Society for Cardiac Angiography and Intervention (SCAI)^39^ proposed that postprocedural cTn≥70 × ULN was “clinically relevant MI”. Since the postprocedural cTn levels reflected the mass of new myocardial injury,^47^ it could be inferred that the prognosis after PCI would positively correlate to the postprocedural cTn levels though there were still not absolute cut‐off values.

In our present study, after adjustment of other covariates excluding lipid profile indices, increased preprocedural Lp(a) was correlated to the elevation of postprocedural cTnI above 1 × ULN up to 15 × ULN but not ≥25 × ULN and ≥ 70 × ULN. The results were similar after adjusted covariates including TG, LDL‐C, non‐HDL‐C and HDL‐C. Therefore, it can be deduced that Lp(a) was correlated to the minor myocardial injury after PCI. Larger mass of myocardial injury, however, was much more strongly correlated to the heart function, complexity of coronary artery lesion and acute damage of PCI procedure as previous article reported.^15^ The major risk factors of PCI‐related myocardial injury could be grouped as lesion‐related, patient‐related and procedure‐related which contributed almost equally. Lp(a) was a patient‐related risk factor. The underlying mechanisms for the association between Lp(a) and minor myocardial injury after PCI remained uncertain yet, as well as the mechanisms for the association between Lp(a) and ASCVD. The structure of Lp(a) has already been illustrated clearly. It was composed of an LDL‐like particle and a specific component apo(a) via a single disulphide bond.^1^ LDL with its apolipoprotein apoB‐100 has already been proven to play the key role in ASCVD.^2^ However, existence of apo(a) made Lp(a) having relatively different physiological and pathological mechanisms to LDL. Similarly to LDL, Lp(a) could be oxidized then phagocytosed by macrophages, making it transform into foam cells and release proinflammatory cytokines.^8^ The proinflammatory effects of Lp(a) were mainly mediated by its component oxidized phospholipids which were covalently bound with apo(a).^9^ Moreover, studies have already showed high Lp(a) levels were linked to the risks of both coronary thrombosis and venous thrombosis.^8^ Because apo(a) moiety and plasminogen was of structural similarities, the reasons why Lp(a) caused thrombosis were speculated to be inhibiting fibrinolysis by interfering with the conversion of plasminogen to plasmin.^8^, ^9^, ^48^ Therefore, besides the atherogenic risk of LDL particles, the proinflammatory and thrombotic effects of Lp(a) might cause the myocardial injury after PCI. However, further definitive mechanisms how Lp(a) promotes atherosclerotic lesions require more appropriate animal models and basic researches to figure out.

As mentioned above, plasma Lp(a) levels were mainly genetically determined,^33^ which were insensitive to lifestyle such as diet and exercise.^8^ Effects of statins on Lp(a) metabolism remained uncertain and controversial.^2^ Therefore, Lp(a) levels were much more stable than LDL‐C. Since there were plenty of evidences demonstrating the association between Lp(a) and ASCVD, it could be expected as a good predictor in the risk of cardiovascular events. Lowering Lp(a) therapies has been developing for these years. Appropriate dose of nicotinic acid which was unique in lowering Lp(a) levels have been showed no beneficial effects.^19^ PCSK9 inhibitors could reduce Lp(a) levels by 25–30% while LDL‐C by 50–60% according to the clinical trials.^9^ The treatment of PCSK9 inhibitors could significantly decrease the ASCVD events but whether it was due to lowering of Lp(a) or simply due to lowering LDL‐C to levels remained controversial.^49^, ^50^, ^51^ Our study revealed the association of elevated Lp(a) levels and risk of PCI‐related myocardial injury but further interventional studies were absent. Therefore, further studies were of expectation to investigate whether lowering of Lp(a) levels could reduce the risk of PCI‐related myocardial injury.

There were still some limitations in this study. First, although this was a multicenter study, the sample size of patients with postprocedural cTnI≥25 × ULN and ≥ 70 × ULN was still relatively small due to the low incidences. Thus the results in postprocedural cTnI≥25 × ULN and ≥ 70 × ULN were not very convincing. Second, as it was a retrospective study, the confounders might be complex. Although we adjusted the factors which might affect postprocedural cTnI levels as many as possible in the multivariable logistic regression analyses, there were still potential confounders might have been not entirely eliminated. Third, our study was an observational study without intervention on Lp(a) levels. Thus although we found the positive correlation between elevated Lp(a) levels and postprocedural cTnI above 1 × ULN up to 15 × ULN, the effects of periprocedural myocardial injury by lowering Lp(a) levels remained unclear. Fourth, since a lot of studies had already investigated the prognostic significance of different elevation of postprocedural cTn levels, we have not conducted further survival analyses of the patients in our study. Whether the postprocedural cTnI elevation could increase the cardiovascular events after PCI of these patients remained uncertain yet.

## 5 CONCLUSION

In non‐AMI CHD patients who successfully underwent PCI, this study found that elevated preprocedural Lp(a) levels were correlated to the risk of postprocedural cTnI greater than 1 × ULN up to 15 × ULN but not 20 × ULN, 25 × ULN and 70 × ULN. As a categorical variable with a cut‐off value 300 mg/L, Lp(a) > 300 mg/L was an independent risk factor of postprocedural cTnI≥1 × ULN, ≥5 × ULN and ≥ 10 × ULN. Therefore, we can draw a conclusion that Lp(a) was associated to the risk of minor myocardial injury after PCI and could be a good predictor of PCI‐related myocardial injury.

## CONFLICT OF INTEREST

The authors have no conflicts of interests.

## AUTHOR CONTRIBUTIONS

Liu Jinlai and Yu Shujie designed the study and were in charge of the overall direction and planning. Huang Zhuoshan analyzed the data and wrote Methods, Results and Discussion part of the manuscript with input from all the authors. Shui Xing performed the statistical analyses. Zhou Linli wrote Abstract and Introduction part of the manuscript. Shi Wenqi, Ling Yesheng and Luo Yanting collected the data needed for our study. Li Suhua and Zhu Jieming were responsible for the modification of manuscript.

## Supporting information


**Supplementary Figure 1** Influence factors of postprocedural cTnI≥25 × ULN and ≥ 70 × ULN explored by multivariate logistic regression analysis. A, Categorical Variables. B, Continuous Variables (Adjusted OR for 1‐SD increment)

**Supplementary Table 1** CAG and PCI Parameters

## Data Availability

The data that support the findings of this study are available from the corresponding author upon reasonable request.
