# Supplementary figures and images for: Serum lipoprotein(a) and risk of periprocedural myocardial injury in patients undergoing percutaneous coronary intervention
Source: Clin Cardiol. 2020 Dec 2;44(2):176–85. doi: 10.1002/clc.23520 (PMC7852163; doi:10.1002/clc.23520)

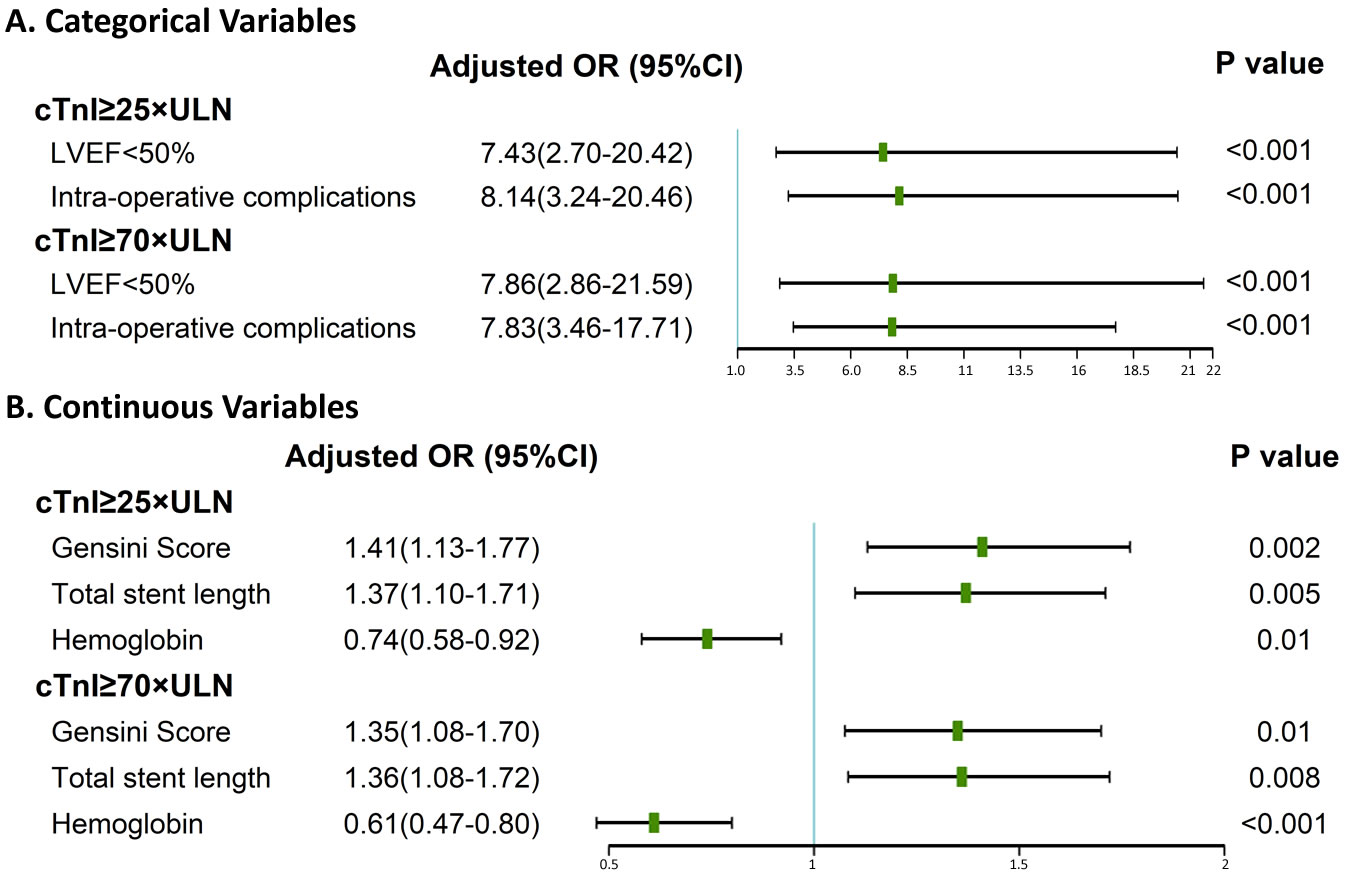

Supplement: Supplementary file 1 — Supplementary Figure 1 Influence factors of postprocedural cTnI≥25 × ULN and ≥ 70 × ULN explored by multivariate logistic regression analysis. A, Categorical Variables. B, Continuous Variables (Adjusted OR for 1‐SD increment) [file CLC-44-176-s001.jpg]
