# Supplementary material for: Serum lipoprotein(a) and risk of periprocedural myocardial injury in patients undergoing percutaneous coronary intervention
Source: Clin Cardiol. 2020 Dec 2;44(2):176–85. doi: 10.1002/clc.23520 (PMC7852163; doi:10.1002/clc.23520)
Supplement: Supplementary file 2 — Supplementary Table 1 CAG and PCI Parameters [file CLC-44-176-s002.docx]

Supplementary Table 1 CAG and PCI Parameters

|  | Lp(a)≤300mg/L  (n=423) | Lp(a)＞300mg/L  (n=105) | P value |
| --- | --- | --- | --- |
| Gensini Score | 41.75±28.49 | 47.58±33.36 | 0.070 |
| Number of intervention vessels | 1.40±0.57 | 1.29±0.62 | 0.067 |
| Intervention vessel location |  |  |  |
| LM, n (%) | 10(2.4) | 4(3.6) | 0.482 |
| LAD, n (%) | 274(65.7) | 63(56.8) | 0.081 |
| LCX, n (%) | 123(29.5) | 37(33.3) | 0.434 |
| RCA, n (%) | 152(36.5) | 33(29.7) | 0.187 |
| OM, n (%) | 11(2.6) | 6(5.4) | 0.142 |
| D1, n (%) | 10(2.4) | 0(0.0) | 0.100 |
| D2, n (%) | 2(0.5) | 0(0.0) | 0.465 |
| PDA, n (%) | 6(1.4) | 0(0.0) | 0.204 |
| Number of stents | 1.67±0.88 | 1.62±0.85 | 0.616 |
| Total stent length, mm | 45.03±29.08 | 44.70±26.97 | 0.918 |
| Maximum inflation pressure, atm | 15.56±2.59 | 15.70±2.94 | 0.864 |
| ACT^a^, s | 232.46±36.27 | 243.67±40.43 | 0.565 |
| Intra-operative Complications, n (%) | 18(4.3) | 6(5.4) | 0.625 |
| Coronary artery dissection, n (%) | 4(1.0) | 3(2.7) | 0.165 |
| Malignant arrhythmia, n (%) | 4(1.0) | 1(0.9) | 0.955 |
| Slow flow, n (%) | 6(1.4) | 1(0.9) | 0.660 |
| Branch artery occlusion, n (%) | 2(0.5) | 1(0.9) | 0.508 |
| Coronary artery spasm, n (%) | 2(0.5) | 0(0.0) | 0.465 |

LM, left main coronary artery; LAD, left anterior descending artery; LCX, left circumflex artery; RCA, right coronary artery; OM, obtusemarginal; D1, first diagonal branch; D2, second diagonal branch; PDA, posterior descending artery; ACT, activated clotting time.

a. ACT was measured 1 hour after PCI finished.
